# Predictors of COVID-19 infection among health care workers in Harare City, Zimbabwe, 2021

**DOI:** 10.11604/pamj.2023.46.76.34014

**Published:** 2023-11-08

**Authors:** Talent Bvochora, Hilda Bara, Addmore Chadambuka, Tsitsi Juru, Mujinga Karakadzai, Prosper Chonzi, Notion Gombe, Mufuta Tshimanga

**Affiliations:** 1 University of Zimbabwe, Department of Primary Health Care Sciences, Global and Public Health Unit, Harare, Zimbabwe,; 2 Harare City Health Department, Harare, Zimbabwe,; 3 African Field Epidemiology Network, Harare, Zimbabwe

**Keywords:** Predictors, COVID-19, health-workers, Harare City, Zimbabwe

## Abstract

**Introduction:**

preventing COVID-19 infection among health workers maintains the health system capacity and reduces secondary transmission. Of 506 health workers tested for severe acute respiratory syndrome coronavirus 2 (SARS-CoV-2) infection in Harare City between December 2020 and February 2021 (second wave), 128 (25%) tested positive, affecting service delivery. We investigated factors associated with COVID-19 infection and described breakthrough infections among health workers.

**Methods:**

we conducted a cross-sectional study in Harare City. Interviews were conducted with 467 health workers to determine behavioral, occupational, and non-occupational factors associated with COVID-19 infection. Fifteen key informants were interviewed to verify responses. Records and line-list were reviewed to verify cases, outcomes, and vaccination status. Observations were done to check adherence to prevention measures. Epi-info generated means, frequencies, proportions and conducted univariate, bivariate and multivariate analysis. Statistical significance was at p-value<0.05.

**Results:**

we interviewed 467 health workers, 166 (35.5%) had a history of COVID-19 infection. Females were the majority 357 (76.4%), mostly nurses 200 (42.8%). Those not trained on infection control measures in the past six months (aOR=2.13; 95%CI 1.06-4.27; p=0.03), not observing social distance at mealtimes (aOR=6.33; 95%CI 3.36-11.89; p<0.01), having a household COVID-19 case (aOR=9.03; 95%CI 3.93-20.76; p<0.01) and not properly wearing facemasks (aOR=16.68; 95%CI 9.39-29.61; p<0.01) were significantly associated COVID-19 infection. Of 378 health workers fully vaccinated, 39 (10.3%) had breakthrough infections. Most with breakthrough infections, 33 (84.6%) had asymptomatic or mild disease. One death was recorded, a female, not vaccinated, with uncontrolled diabetes.

**Conclusion:**

predictors of COVID-19 infection among health workers were, no training on infection and prevention and control (IPC) measures, lack of social distancing at mealtimes, improper use of facemasks, and having a COVID-19 case at household level. We conducted refresher training to strengthen infection control measures.

## Introduction

Coronavirus disease 2019 (COVID-19), is a pandemic declared by the World Health Organization (WHO) in March 2020 [1]. It is a highly infectious respiratory disease caused by the severe acute respiratory syndrome coronavirus 2 (SARS-CoV-2) [2]. Transmission in humans occurs when people are in close contact with one another through respiratory droplets produced when an infected person coughs or sneezes. The virus remains on several surfaces for some time, hence it may be possible that a person can get coronavirus disease of 2019 (COVID-19) by touching a surface or object that has the virus on it and then touching their mouth, nose, or eyes [3]. Studies have suggested that SARS-CoV-2 may be transmitted by both symptomatic and asymptomatic positive cases [4]. Globally as of May 24, 2021, there had been 167,252,150 confirmed cases of COVID-19, including 3,467,663 deaths [5]. Zimbabwe had 38,696 confirmed cases, including 36,471 recoveries and 1,586 deaths recorded as of the 24^th^ of May 2021 and the cumulative statistics for Harare were 11,904 confirmed cases, 915 deaths, and 10,918 recoveries [6]. As of 20^th^ May, 152,106 received dose 1, and 81,059 received dose 2, of the COVID-19 vaccine in Harare [7].

Health care workers (HCWs) are on the frontline in fighting the COVID-19. World Health Organization (WHO) defines health workers as people whose job is to protect and improve the health of their communities [8]. This comprises doctors, nurses, paramedical staff, hospital administrators, and other support staff. The critical role played by healthcare workers in fighting the COVID-19 pandemic puts them at a higher risk of COVID-19 infection than the general community [9,10]. To minimize healthcare worker exposure to the virus there is a need for training on infection and prevention and control (IPC), provision of the IPC resources, and monitoring their use [11]. It has been reported that correct and consistent compliance with IPC protocols is effective in minimizing the risk of COVID-19 infection [9,10]. In general, IPC strategies in response to highly infectious diseases, such as COVID-19, should include early recognition, source control, physical distancing, taking precautions and appropriate use of personal protective equipments (PPEs), restriction of movement, environmental cleaning, and disinfection as well as support for healthcare workers [11].

Zimbabwe has experienced two waves of the pandemic and healthcare workers have not been spared with a total of over 5000 (11%) HCWs infected by COVID-19 as of May 2021 [12]. The second wave of COVID-19 infections in Harare started in mid-December 2020 [12]. Of the 506 healthcare workers tested for SARS-CoV-2 infection using the polymerase chain reaction (PCR) test in Harare City facilities between December 2020 and February 2021, 128 (25%) tested positive for the viral infection. Unlike in the first wave where five health facilities in the city had positive cases among healthcare workers, in the second phase 15 health facilities were affected. Infected healthcare workers and their colleagues who were contacts had to go for isolation and quarantine respectively negatively affecting service delivery in the city. These infections occurred despite there being adequate personal protective clothing and trainings on infection prevention and control. With the current rise of infections and a scare of a third wave, we, therefore, saw the need to investigate factors associated with COVID-19 infection in the city so we can give recommendations on HCW protection.

## Methods

**Study design:** an analytic cross-sectional study was conducted.

**Study setting:** Harare City Health Department is run under Harare Municipal Authority as a parastatal to the Government of Zimbabwe. The department has two infectious diseases hospitals (Wilkins and Beatrice Road), an emergency services center, and 43 clinics which are distributed in its four districts. There are currently 1013 healthcare workers in the city. There are 13 health facilities that had HCWs who contracted COVID-19 from June 2020 to June 2021. The study was carried out at Harare City health facilities that had healthcare workers who contracted COVID-19.

**Study population:** the study population consisted of HCWs tested for COVID-19 using the PCR test in Harare City. These include doctors, nurses, nurse aides, dental personnel, laboratory personnel, primary councillors, pharmacy personnel, environmental health cadres, general hands, clerks, and administrative personnel.

### Operational definition

**COVID-19 infection:** in this study, COVID-19 infection was defined as a positive PCR or a rapid antigen test for COVID-19 at any period during the pandemic.

**Vaccinated:** in this study, those said to be vaccinated were those who had received two doses of the vaccine. Those who had breakthrough infection were those who contracted COVID-19 two weeks after having received the second dose of the vaccine.

**Sample size calculation:** sample size was calculated using Fleiss formula and based on a study by Sokkary *et al*. “Characteristics and predicting factors of coronavirus disease-2019 (COVID-19) among healthcare providers in a developing country”. Assuming 80% power at 95% CI, a precision of 5% with a prevalence of 20% COVID-19 infection among HCWs adhering to IPC measures, odds ratio (OR) = 2.1, a minimum sample size of 460 was calculated.

**Sampling:** all health facilities reported to have HCWs who contracted COVID-19 in Harare City were included in the study. All healthcare workers found on duty at facilities, had been tested for COVID-19 within the month, and were willing to participant were included.

**Data collection and tools:** an interviewer-administered questionnaire was used to collect data on demographic, behavioural, occupational, and non-occupational factors associated with COVID-19 infection among healthcare workers. Study participants were interviewed at their workstations. Records and the line list were reviewed to verify those who tested positive and on vaccination status. Observations were done to check on adherence to IPC measures.

**Data analysis:** an electronic template was created, and data was captured and analyzed using Epi Info 7.2.4.0 ™ (The Centers for Disease Control and Prevention (CDC), 2020) statistical software. Data was entered, and cleaned for transcription errors, missing details, duplicate information, and values that are out of range. Descriptive summaries were generated for all study variables using means, medians, and confidence intervals for quantitative variables and frequencies and percentages for categorical variables. Bivariate analysis was used to determine the strengths of the association between the independent variables and the outcome variable (COVID-19 status). Crude odds risks (OR) and 95% confidence intervals were calculated. Statistical significance was considered at a p-value <0.05 and only significant variables were entered into the logistic regression model. Multivariate logistic regression was used to identify factors independently associated with COVID-19 infection. Adjusted odds ratios (aOR) and 95% confidence intervals were calculated.

**Permission and ethical considerations:** permission to carry out the study was obtained from the City of Harare Institutional Review Board (IRB) and the Joint Research Ethics Committee (JREC) at the University of Zimbabwe (ref 345/2021). Written informed consent was obtained from the study participants. Confidentiality was assured and maintained throughout the study. Each participant was interviewed privately, and we ensured that the information obtained was not disclosed to any persons other than those relevant for the purposes of the study. All questionnaires are kept under lock and key. Wearing of face masks, social distancing, and hand sanitizing was observed.

## Results

**Socio-demographic characteristics of HCWs tested for COVID-19 in Harare City:** a total of 467 healthcare workers were interviewed of which 166 (35.5%) had a history of having contracted COVID-19. Females were the majority 357 (76.4%) and constituted 135 (81.3%) of those who contracted COVID-19. Most of the workers were nurses 200 (42.8%). Of the 166 HCWs who contracted COVID-19, 66 (39.8%) were nurses. The median age of the HCWs who contracted COVID-19 was 41 (Q_1_= 35, Q_3_= 48) years and their median years in service was 10 years (Q_1_= 3, Q_3_= 20). The majority who contracted COVID-19, 88/166 (53.0%) had worked for 10 or less years for Harare City. Details of socio-demographic factors are in Table 1. No socio-demographic factors were statistically significant determinants of SARS-CoV-2 among HCWs in the City of Harare as shown in Table 1.

**Table 1:** demographic factors associated with COVID-19 infection among health care workers (HCWs) in Harare City, 2021

Variable	Category	COVID-19 positive n=(%)	COVID-19 negative n=(%)	OR	95 % CI	P value
Sex	Female	135 (81.3)	222 (73.7)	1.55	0.97-2.47	0.06
	Male	31 (18.7)	79 (26.3)			
Age	≤ 40	84 (50.6)	136 (45.2)	1.24	0.85-1.82	0.26
	>40	82 (49.4)	165 (54.8)			
Median age		40 (Q^1^= 35, Q^3^= 48)	42 (Q^1^= 33, Q^3^= 50)			
Level of education	Tertiary	105 (63.3)	190 (63.1)	1.01	0.67-1.49	0.98
	Below tertiary	61 (36.7)	111 (36.9)			
Marital status	Married	118 (71.1)	199 (66.1)	1.26	0.83-1.90	0.27
	Not married	48 (28.9)	102 (33.9)			
Designation	Clinicians	125 (75.3)	228 (76.3)	0.95	0.61-1.48	0.82
	Non clinicians	41 (24.7)	71 (23.8)			
Years in service	≤ 10	88 (53.0)	167 (55.5)	0.91	0.62-1.32	0.61
	>10	78 (47.0)	134 (44.5)			
Median years in service		10 (Q^1^= 3, Q^3^= 20)	10 (Q^1^= 3, Q^3^= 19)			
Religion	Pentecostal	52 (31.33)	110 (36.5)	0.79	0.52-1.19	0.26
	Non-pentecostal	114 (68.8)	191 (63.5)			

**Behavioural factors associated with COVID-19 infection among HCWs in Harare City:** using univariate analysis, the behavioural factors that were found to be associated with COVID-19 infection were not washing hands (OR=10.4, 95%CI 6.46-16.63; p<0.01), not properly using face masks (OR=25.1; 95%CI 15.2-42.6; p<0.01), and not practicing social distancing at mealtimes (OR= 12.15, 95%CI 7.2-20.47; p<0.01). Regular exercising (OR=0.50, 95%CI 0.35-0.75; p<0.01) and being vaccinated (OR=1.94, 95%CI 1.21-3.10; p<0.01) were found to be protective against contracting COVID-19. Table 2 shows details of behavioural factors associated with COVID-19 infection among HCWs in Harare City.

**Table 2:** behavioural factors associated with COVID-19 infection among health care workers (HCWs) in Harare City, Zimbabwe 2021

Variable	Category	COVID-19 positive n=(%)	COVID-19 negative n=(%)	OR	95 % CI	P value
Vaccination status	No	43 (25.9)	46 (15.2)	1.94	1.21-3.10	0.005
	Yes	123 (74.1)	255 (84.7)			
Regular physical exercise	No	115 (69.3)	160 (53.2)	2.00	1.33-2.96	<0.01
	Yes	51 (30.7)	141 (46.8)			
Washing hands	Sometimes	138 (83.1)	97 (32.2)	10.4	6.46-16.63	<0.01
	All the time	28 (16.9)	204 (67.8)			
Social distancing at meal times	Sometimes	146 (88.0)	113 (37.5)	12.15	7.20-20.47	<0.01
	All the time	20 (12.0)	188 (62.5)			
Proper use of face mask	Sometimes	131 (78.9)	39 (13.0)	25.1	15.2-41.6	<0.01
	All the time	35 (21.1)	262 (87.0)			
Current tobacco use	Yes	4 (2.4)	11 (3.7)	0.65	0.20-2.08	0.47
	No	162 (97.6)	290 (96.3)			
Current alcohol use	Yes	36 (21.7)	69 (22.9)	0.93	0.59-1.47	0.76
	No	130 (78.3)	232 (77.1)			
Regular physical exercise	Yes	51 (30.7)	141 (46.8)	0.50	0.34-0.75	<0.01
	No	115 (69.3)	160 (53.2)			
Vaccination status	Yes	123 (74.1)	255 (84.7)	0.52	0.32-0.82	0.005
	No	43 (25.9)	46 (15.3)			

**Occupational and non-occupational factors associated with COVID-19 infection among HCWs in Harare City:** occupational factors that were significantly associated with COVID-19 infection among HCWs were PPE non-availability (OR=1.70; 95%CI 1.11-2.59; p=0.01), not having been trained on infection control and prevention and use of personal protective equipment (OR= 2.53; 95%CI 1.51-4.24; p=0.0002), and non-availability of a handwashing facility at the workstation (OR=3.82; 95%CI 2.56-5.69; p<0.01.). Working at the outpatients was found to be protective against contracting COVID-19 (OR=0.63, 95% CI 0.43-0.92; p=0.02) as in Table 3. PPE was readily available at all facilities visited. The number of face masks given varied between clinicians and non-clinicians from 3-5 per day and 1-2 per day respectively. Non-occupational factors that were significant predictors of COVID-19 were the size of household members the HCWs lived with (OR=1.92, 95%CI 1.28-2.89; p<0.01) and if there was a COVID-19 case at home (OR=9.07, 95%CI 4.99-16.50; p<0.01). Details of non-occupational factors are in Table 3.

**Table 3:** occupational, non-occupational and independent factors associated with COVID-19 infection among health care workers (HCWs) in Harare City, Zimbabwe 2021

Variable	Category	COVID-19 positive n=(%)	COVID-19 negative n=(%)	OR	95 % CI	P value
Availability of PPE	No	55 (33.1)	68 (22.6)	1.70	1.11- 2.59	0.01
	Yes	111 (66.8)	233 (77.4)			
Training on IPC/PPE use	No	144 (86.8)	217 (72.1)	2.53	1.51- 4.24	0.0003
	Yes	22 (13.2)	84 (27.9)			
Availability of hand washing facilities	No	99 (59.6)	84 (27.9)	3.82	2.56- 5.69	<0.01
	Yes	67 (40.4)	217 (72.1)			
Workplace-wards	Yes	54 (32.5)	81 (26.9)	0.76	0.51- 1.15	0.20
	No	112 (67.5)	220 (73.1)			
Outpatients	Yes	75 (45.2)	171 (56.8)	0.63	0.43- 0.92	0.02
	No	91 (54.8)	130 (43.2)			
Office	Yes	26 (15.7)	35 (11.6)	0.71	0.41- 1.22	0.22
	No	140 (84.3)	266 (88.4)			
Outdoors	Yes	11 (6.6)	14 (4.7)	1.45	0.64- 3.28	0.36
	No	155 (93.4)	287 (95.3)			
Working hours	>8 hours	49 (29.5)	78 (25.9)	1.20	0.79- 1.83	0.40
	≤8 hours	117 (70.5)	223 (74.1)			
Support and supervision	No	99 (59.6)	156 (51.8)	1.37	0.94- 2.01	0.10
Size of household	>4	119 (71.7)	171 (56.8)	1.92	1.28- 2.89	0.002
	≤4	47 (28.3)	130 (43.2)			
COVID-19 case at home	Yes	56 (33.7)	16 (5.3)	9.07	4.99- 16.5	<0.01
	No	110 (66.3)	285 (94.7)			
Means of transport	Public	119 (71.7)	197 (65.5)	1.34	0.88- 2.02	0.17
	Private	47 (28.3)	104 (34.6)			
**Independent factors associated with COVID-19 infection among HCWs in Harare City, Zimbabwe 2021**						
**Variable**		**aOR**			**95 % CI**	**P value**
No training in past six months		2.13			1.06- 4.27	0.03
No social distance at meal times		6.33			3.36- 11.89	<0.01
Covid-19 case at home		9.03			3.93- 20.76	<0.01
Not properly wearing face mask		16.68			9.39- 29.61	<0.01

PPE: personal protective equipment; IPC: infection and prevention and control

**Independent factors associated with COVID-19 infection among HCWs in Harare City:** independent factors found to be significantly associated with COVID-19 infection were not having had training on infection and prevention control measures in the past six months (aOR=2.13; 95%CI 1.06-4.27; p=0.03), not observing social distance at mealtimes (aOR=6.33; 95%CI 3.36-11.89; p<0.01), having a COVID-19 case at home (aOR=9.03; 95%CI 3.93-20.76; p<0.01).and not properly wearing face masks (aOR=16.68; 95%CI 9.39-29.61; p<0.01) as shown in Table 3.

**COVID-19 vaccination coverage among HCWs in Harare City:** overall vaccine coverage was high with a total of 378 (80.9%) HCWs having been vaccinated by September 2021. Coverage among clinicians (nurses, primary councillors, environmental health technicians and nurse aides) was high, save for doctors and dental personnel. The lowest coverage was among laboratory personnel, 5/12 followed by pharmacy, dental and administrative personnel, all at 66.7% coverage.

**COVID-19 vaccination status among HCWs who contacted the viral disease:** health care workers who got COVID-19 infected before the rollout of vaccination were 90/166 (54.2%). Of those who contracted COVID-19 after vaccination had started, 39/76 (51.3%) were fully vaccinated and 37/76 (48.7%) were not vaccinated.

**COVID-19 infection outcomes among HCWs in Harare City:** of the 166 HCWs who had COVID-19 infection, 133 (80.1%) had mild symptoms and recovered within 14 days of isolation. Fifteen HCWs (9.0%) were in isolation for 15-21 days and had mild disease. Of the five health care workers who were admitted into the hospital for severe disease, 4 recovered and one died. Eighteen HCWs (10.8%) who had moderate to severe disease were treated at home and recovered.

**Breakthrough infections among HCWs in Harare City:** of the 378 HCWs who had been fully vaccinated, there were 39 (10.3%) breakthrough infections. Most of the HCWs 33 (84.6%) who had breakthrough infections, had mild disease with flu-like symptoms. None of them had persistent symptoms of more than six weeks. One death among healthcare workers was recorded, of a female who had uncontrolled diabetes and was not vaccinated.

## Discussion

The study findings were that behavioural factors which included social distancing at mealtimes and properly wearing face masks by health care workers were protective against COVID-19 infection. Those who had received training in the past six months on COVID-19 infection prevention and control were less likely to contract the infection. However, having a household member diagnosed with COVID-19 was a risk factor for COVID-19. Vaccine coverage among health care workers was high.

With the nature of the disease and how it is spread, COVID-19 is likely to spread among people with close relations. In households it has been noted that most people do not wear any protective clothing nor do they social distance. Transmission of COVID-19 can hence easily occur in these spaces. Having had a COVID-19 case at home could have meant one of two things, that the HCW infected the household members or that the household members infected the HCW. In the community, and especially in a household, both HCWs and their families do not commonly practice IPC measures and if any one of them were to contract COVID-19, it is easily transmitted in that household. The CDC noted that infection can be transmitted from pre-symptomatic cases to HCWs not adhering to IPC measures both at the workplace and at home [13]. This is consistent with our findings where COVID-19 infection at the household was a predictor of infection among the HCWs.

Health care workers usually go to mealtimes at specific times. Sitting spaces in dining facilities were not at least one meter apart hence it was not possible to practice social distancing if in full capacity. For health care workers to social distance at mealtimes, there was, therefore, a need to stagger the mealtimes so that social distancing could be achieved. It was observed however, that there were workstations and facilities where health care workers were going for meals times at the same time and spending more than 15 minutes without wearing masks and did not maintain social distance from their colleagues. This could have led to an increased risk of them being infected. Prolonged, unprotected gatherings with colleagues, such as meal times have also been shown by Sokarry *et al*. as a risk factor to infection with COVID-19 [14].

Face masks and other personal protective clothing such as gowns and face shields were readily available at all facilities. The number of face masks that were being distributed per HCW per day depended on whether one was a clinician or not. Support staff members such as general labourers were given only one mask per day at most facilities, and this meant they were not changing their masks the whole working period. Key informants also reported that HCWs do not change their masks as frequently as they should as they were keeping them for their family members at home. This can also lead to contamination of the masks and increase the chance of getting COVID-19 infected. It was observed that health care workers tended to lower their face masks and leave their noses exposed. This might have led to contracting COVID-19 if there was touching of the nose after getting into contact with either a patient or a surface that was contaminated with COVID-19 [15]. Some who worked in closed spaces such as the pharmacy and laboratory were observed to completely remove their masks when in these areas. They perceived that they were at low risk of contracting COVID-19 and some reported having difficulties in wearing masks for long periods.

At the beginning of the pandemic, training in infection prevention and control measures for COVID-19 were done and most HCWs were trained. However, refresher training, support, and supervision on COVID-19-related issues had not been done for more than six months at most of the health facilities. Those who had received training within six months before data collection could remember the IPC measures and the appropriate PPE to put on hence putting them at a lower risk of contracting COVID-19. Lack of training or a long period following training could have led to HCWs forgetting the IPC measures needed to be observed or made HCW complacent hence exposing them to a greater risk of being infected. Unlike in our study where lack of or late training of HCWs and not properly wearing face masks was associated with infection, a study by Al Abri *et al*. showed that while the majority of HCWs followed crucial IPC measures, one-third had never received specific IPC training [16]. However similar to our study, inadequate training and poor adherence to IPC measures were predictors of COVID-19 infection among HCWs in various studies [14,17].

Vaccine coverage was generally high due to the massive, individualized campaigns done as reported by key informants. Discrepancies in vaccination coverage seen among the different cadres were probably due to non-clinicians presuming that they are at low risk of contracting COVID-19. This, however, is in contrast with a study by CDC which showed that nurses had lower vaccine coverage [18]. Breakthrough infections presented with mild disease as was noted in other studies [19,20].

**Limitations:** a limitation to our study was recall bias as some of the HCWs contracted COVID-19 more than six months before the study and could not recall events leading to their infection. We, therefore, used observations, key informant responses, and records to support the responses given.

## Conclusion

Our study identified the predictors of COVID-19 infection among health care workers as training on IPC measures, social distancing at mealtimes, proper use of PPE, and having a COVID-19 case at the household level. Vaccination coverage among the health care workers was generally high at 81%. Breakthrough infections presented with mild disease. There is a need therefore for vaccine promotion through education and training resources would improve the coverage.

### What is known about this topic

- *What causes COVID-19 and how it is spread is known, however, new information keeps being discovered as it is a novel pandemic*.

### What this study adds

- *The study adds supportive evidence on the predictors of COVID-19 among health care workers, namely social distancing at mealtime, proper wearing of protective equipment and training on infection prevention and control;*
- *The study adds that breakthrough COVID-19 infections present with mild to moderate symptoms*.
